# Neuropilin-2 expression in breast cancer: correlation with lymph node metastasis, poor prognosis, and regulation of CXCR4 expression

**DOI:** 10.1186/1471-2407-9-220

**Published:** 2009-07-07

**Authors:** Hironao Yasuoka, Rieko Kodama, Masahiko Tsujimoto, Katsuhide Yoshidome, Hiroki Akamatsu, Masaaki Nakahara, Michiya Inagaki, Tokio Sanke, Yasushi Nakamura

**Affiliations:** 1 Department of Clinical Laboratory Medicine, Wakayama Medical University, Wakayama, Japan; 2 Department of Pathology, Osaka Police Hospital, Osaka, Japan; 3 Department of Surgery, Osaka Police Hospital, Osaka, Japan

## Abstract

**Background:**

Neuropilin-2 (Nrp2) is a receptor for vascular endothelial growth factor-C (VEGF-C), which is a well-known lymphangiogenic factor and plays an important role in lymph node metastasis of various human cancers, including breast cancer. Recently, Nrp2 was shown to play a role in cancer by promoting tumor cell metastasis. CXC chemokine receptor 4 (CXCR4) also promotes tumor metastasis. In the previous studies, we demonstrated that VEGF-C and cytoplasmic CXCR4 expressions were correlated with poorer patient prognosis (BMC Cancer 2008,8:340; Breast Cancer Res Treat 2005, 91:125–132).

**Methods:**

The relationship between Nrp2 expression and lymph node metastasis, VEGF-C expression, CXCR4 expression, and other established clinicopathological variables (these data were cited in our previous papers), including prognosis, was analyzed in human breast cancer. Effects of neutralizing anti-Nrp2 antibody on CXCR4 expression and chemotaxis were assessed in MDA-MB-231 breast cancer cells.

**Results:**

Nrp2 expression was observed in 53.1% (60 of 113) of the invasive breast carcinomas. Nrp2 expression was significantly correlated with lymph node metastasis, VEGF-C expression, and cytoplasmic CXCR4 expression. Survival curves determined by the Kaplan-Meier method showed that Nrp2 expression was associated with reduced overall survival. In multivariate analysis, Nrp2 expression emerged as a significant independent predictor for overall survival. Neutralizing anti-Nrp2 antibody blocks cytoplasmic CXCR4 expression and CXCR4-induced migration in MDA-MB-231 cells.

**Conclusion:**

Nrp2 expression was correlated with lymph node metastasis, VEGF-C expression, and cytoplasmic CXCR4 expression. Nrp2 expression may serve as a significant prognostic factor for long-term survival in breast cancer. Our data also showed a role for Nrp2 in regulating cytoplasmic CXCR4 expression *in vitro*.

## Background

Neuropilin-2 (Nrp2) is a transmembrane glycoprotein that interacts with vascular endothelial growth factor (VEGF). Nrp2 is involved in the regulation of many physiological conditions, including angiogenesis [1]. Previous reports have suggested a possibility that Nrp may function independently of VEGF receptors to modulate endothelial cell migration, which may lead to angiogenesis [2,3]. Nrp2 is also known to function as a receptor for VEGF-C [4,5], which is a well-known lymphangiogenic factor and plays an important role in lymph node metastasis of various human cancers [6], including breast cancer [7]. Recently, Nrp2 was shown to play a role in cancer by promoting migration of breast cancer cells, which correlates with tumor cell metastasis [8,9]. However, the role of Nrp2 in human breast cancer is largely unknown.

Metastasis is the major cause of death associated with solid tumors [10]. Metastasis of cancer cells is a complex process including invasion, hemangiogenesis, lymphangiogenesis, trafficking of cancer cells through blood or lymph vessels, extravasations, organ-specific homing, and growth. Recent evidence suggests that CXC chemokine receptor 4 (CXCR4) plays a critical role in the homing of cancer cells to specific metastatic sites [11]. The CXCR4 ligand, CXCL12, was found to be expressed in liver, bone marrow, lung, and lymph nodes, and also shown to promote migration of cancer cells [12]. Previous reports demonstrated that up-regulated CXCR4 expression in human cancers, including breast cancer, was correlated with lymph node metastasis and unfavorable prognosis, and was regulated by nitric oxide [13-15].

In this study, we examined the relationship of Nrp2 to lymph node metastasis, VEGF-C expression, and CXCR4 expression in human breast cancer tissues, and further investigated the potential value or relevance of Nrp2 for predicting disease outcome. We then showed that an antibody against Nrp2 reduces cytoplasmic CXCR4 expression and inhibited CXCL12-induced chemotaxis in MDA-MB-231 breast cancer cells, which were previously shown to express endogenous Nrp2 [8].

## Methods

### Patients and tumor samples

This study was approved by the review board of the Wakayama Medical University Medical Ethics Committee and informed consent was obtained from each of the patients. Archival paraffin-embedded specimens of invasive breast cancer from 113 patients, who were diagnosed and treated between 1981 and 1992 at the Osaka Police Hospital, Japan, were selected as described previously [16]. None of these cases had a family history of breast cancer or malignancy in first-degree relatives as determined by questioning at the time of admission for surgery. The patients had received mastectomy with axillary lymph node dissection. All women were apparently free of distant metastasis. All cases received post-operative adjuvant therapy consisting of combination chemotherapy and hormone treatment. Immunostaining for estrogen receptor (ER), progesterone receptor (PgR), VEGF-C, and CXCR4 had been already performed and the results had been previously described [14,17]. Positivity was defined as nuclear staining of 10% or more cancer cells with a strong intensity for ER and PgR. VEGF-C immunoreactivity was defined as the cases in which at least 10% of tumor cells were immunoreactive. The intensity, staining percentage, and pattern of staining (nuclear and cytoplasmic) were assessed for CXCR4. The intensity was scored as low, moderate, and strong compared with background staining. The percentages of positive cells were estimated by calculating the ratio of the positively stained invasive tumor cells to the total invasive cells. Nuclear versus cytoplasmic location of expression was also noted in each sample. The staining patterns of tumors for CXCR4 was defined as high cytoplasmic expression (moderate and > 50%, or strong and > 30% cytoplasmic expression) or predominantly nuclear expression (at least 80% nuclear expression). The size of the primary tumor was determined from the surgical specimen. Lymph node metastasis was determined by counting the number of axillary lymph nodes with histological evidence of metastatic breast carcinoma. Histological typing and grading were done according to the WHO classification [18] and the Nottingham method (Bloom Richardson) [19]. Patient and tumor characteristics are shown in Table 1. The median age of the 113 patients at diagnosis was 51 years (range, 24–87 years). Fifty-eight percent of the patients were younger than 50 years (n = 65), and 52% (n = 59) of the patients had lymph node metastasis at the time of surgery. Twenty-six percent of the patients had distant organ metastasis during the follow-up period (n = 29).

**Table 1:** The relationship between Neuropilin-2 expression and other parameters.

		Neuropilin-2		p value
Factor		Negative	Positive	

Age	< 50	35 (54%)	30 (46%)	0.0911
	≧51	18 (38%)	30 (62%)	
Histology	Ductal	47 (45%)	57 (55%)	0.4328
	Lobular	3 (60%)	2 (40%)	
	Medullary	1 (100%)	0 (0%)	
	Mucinous	2 (67%)	1 (33%)	
Tumor size	pT1	19 (53%)	17 (47%)	0.4240
	pT2–4	34 (44%)	43 (56%)	
Lymph node metastasis	Negative	31 (57%)	23 (43%)	0.0389
	Positive	22 (37%)	37 (63%)	
Estrogen receptor	Negative	22 (49%)	23 (51%)	0.8476
	Positive	31 (46%)	37 (54%)	
Progesterone receptor	Negative	24 (47%)	27 (53%)	1.0000
	Positive	29 (47%)	33 (53%)	
Histological grade	I and II	34 (51%)	33 (49%)	0.3440
	III	19 (41%)	27 (59%)	
VEGF-C	Negative	17 (85%)	3 (15%)	< 0.001
	Positive	36 (39%)	57 (61%)	
CXCR4 (cytoplasmic)	Negative	37 (65%)	20 (35%)	< 0.001
	Positive	16 (29%)	40 (71%)	
CXCR4 (nuclei)	Negative	42 (50%)	42 (50%)	0.2879
	Positive	11 (38%)	18 (62%)	
Distant metastasis	Negative	41 (49%)	43 (51%)	0.5243
	Positive	12 (41%)	17 (59%)	

### Immunohistochemistry

For immunostaining, 4-micrometer thick paraffin sections were de-paraffinized, placed in a solution of 97% methanol and 3% hydrogen peroxide for 5 min, then autoclaved for antigen retrieval. After washing in phosphate-buffered saline (PBS), the slides were treated for 20 min with Protein Block Serum-free (DAKO Cytomation, Carpinteria, CA, USA). This was followed by an overnight incubation at 4°C in a humidified chamber with a 1:20 diluted goat anti-human Nrp2 antibody (R & D Systems, Inc, Minneapolis, MN, USA). After the overnight treatment, Histofine SAB-PO (NICHIREI, Tokyo, Japan) was used as the second antibody, according to the manufacturer's instructions. Color was developed using diaminobenzidine with 0.01% hydrogen peroxide, and hematoxylin was used as a counterstain. For the negative control, all reagents except for the primary antibody were used. The immunohistochemical scoring was performed blindedly by 3 pathologists (HY, RK, and YN), who had no clinical knowledge of the patients. The immunostained sections of two representative slides of each case were examined by light-microscopy, and all tumor areas were analyzed for Nrp2 positivity, which was defined as the cases in which at least 10% of tumor cells were immunoreactive.

### Cell culture

The MDA-MB-231 breast carcinoma cell line was purchased from the American Type Culture Collection (ATCC, Rockville, MD, USA). MDA-MB-231 cells were maintained at 37°C in 95% air and 5% CO_2_, as monolayers in tissue culture dishes containing DMEM medium (Invitrogen, Tokyo, Japan) supplemented with 10% heat-inactivated fetal calf serum (FCS) (HyClone, South Logan, UT, USA). For the experiments, 6 cm tissue culture plates (Corning Inc, Corning, NY, USA) were seeded with 5 × 10^5 ^cells in 3 mL of medium + 10% FCS. Next day, media was changed to FCS-free medium, and then neutralizing anti-Nrp2 antibody (final concentration was 3 microgram/mL) was added. This concentration of Nrp2 antibody had no effect on cell viability as measured by the CellTiter 96 Aqueous One Solution Cell Proliferation Assay (Promega, Madison, WI, USA) (data not shown).

### Determination of CXCR4 protein expression

For the determination of CXCR4 protein expression, MDA-MB-231 cell lines were incubated for 12 hours with or without neutralizing anti-Nrp2 antibody, and then harvested. Cell lysates were prepared using NE-PER™ Nuclear and Cytoplasmic Extraction Reagents (Pierce, Rockford, IL, USA) containing Halt™ Protease Inhibitor Cocktail (Pierce). For Western blot analysis of CXCR4, 40 microgram samples of nuclear extracts or cytoplasmic fractions were separated by electrophoresis in 10–20% SDS polyacrylamide gels, and transferred to PVDF membranes by electroblotting as described previously [16]. The membrane was blocked with 5% skim milk in PBS for 1 h at room temperature, incubated overnight with anti-human CXCR4 rabbit antibody (Abcam Ltd., Cambridge, UK), washed with PBS, and labeled with peroxidase-conjugated anti-rabbit secondary antibody (Dako Cytomation, Denmark) for 1 h at room temperature. The signals were visualized using the LumiGLO Reserve chemiluminescence substrate kit (KPL, Inc, Gaithersburg, MD, USA) and recorded by luminocapture (ATTO, Tokyo, Japan). Anti-beta-2-microglobulin antibody (Dako Cytomation) was used for the internal control of cytoplasmic extract. Anti-transcription factor IID antibody (Upstate, Lake Placid, NY, USA) was used for the internal control of nuclear extract.

### Chemotaxis assay

Migration assay was performed in 24-well plates using inserts with 8 micrometer pore size membranes (BD Biosciences, Bedford, MA, USA). The surface of the membranes was precoated with 50 microliter of fibronectin (BD Biosciences) in PBS (25 microgram per mL) per filter, allowed to dry at room temperature, and washed with PBS. MDA-MB-231 cells were suspended at 1 × 10^6 ^cells/mL in FCS-free medium and placed in the upper chambers of the membrane. At the same time, the lower compartment of the chamber was loaded with or without 400 ng/mL of recombinant human CXCL12 (R&D Systems), and the upper compartment of the chamber was placed with or without neutralizing anti-Nrp2 antibody (final concentration was 3 microgram/mL). After incubation for 24 hours, the chamber was removed, and cells that had migrated to the bottom of the membrane were fixed, stained in Cyto Quick Solution (Muto Pure Chemical, Tokyo, Japan), and counted by light microscopy. All assays were done in triplicate.

### Statistical analysis

Fisher's exact test was used to examine the association of various clinicopathological factors including Nrp2, VEGF-C expression, and cytoplasmic or nuclear CXCR4 expression. Overall survival (OS) curves and disease-free survival (DFS) curves were obtained using the Kaplan-Meier method and compared using the log-rank test. A multivariate model using the Cox stepwise regression analysis was used to evaluate the statistical strength of independent association between covariates and DFS and/or OS. The effects of neutralizing anti-Nrp2 antibody treatment were analyzed by Student's *t *test. A p value less than 0.05 was considered significant. A software package (JMP IN 5.1.1, SAS Institute, Cary, NC, USA) was used for all statistical testing and management of the database.

## Results

### Nrp2 expression in breast cancer tissue

**Figure 1:**
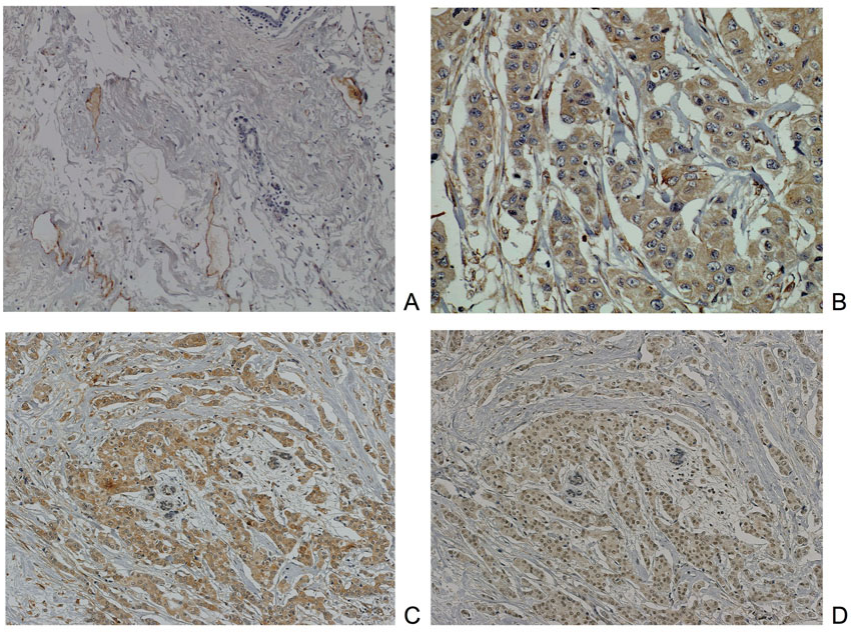
**Neuropilin-2 (Nrp2) expression in normal breast and breast carcinoma tissue**. (A) Nrp2 staining was observed in blood or lymph vessels. There was no staining in normal breast epithelium. (×100). (B) In cancer tissue, staining of the Nrp2 protein was identified not only in the vascular endothelial cells, but also in the cytoplasm of cancer cells. Almost all invasive cells were immunopositive for Nrp2. (×200). (C-D) Co-localized expression of Nrp2 (C) and CXCR4 (D) using serial sections of breast carcinoma tissue. (×100).

In normal breast tissue (Figure 1A), Nrp2 staining was observed in blood or lymph vessels, while there was no staining in normal breast epithelium. In cancer tissue, staining of the Nrp2 protein was identified not only in the vascular endothelial cells, but also in the cytoplasm of cancer cells. In some cases, almost all invasive cells were immunopositive for Nrp2 (Figure 1B). Nrp2 expression was identified in both marginal and central areas of the tumor. According to the criteria for Nrp2 immunostaining evaluation, Nrp2 protein was positive in 53.1% (60 of 113) of the invasive breast carcinomas.

### Nrp2 expression is correlated with lymph node metastasis, VEGF-C expression, and cytoplasmic CXCR4 expression

As we described previously [17], the expression of VEGF-C protein was observed as diffuse cytoplasmic staining in breast cancer cells. VEGF-C expression was positive in 82% (93 of 113) of the breast cancer patients. Nrp2 expression tended to be co-localized or adjacent to VEGF-C expression (data not shown). In the previous study on immunostaining of CXCR4, cytoplasmic staining had been prominent than nuclear staining [14]. Cytoplasmic staining with a nuclear component had been observed in 20% (23 of 113) of those tumors, predominantly nuclear staining in 5% (6 of 113), predominantly cytoplasmic staining in 29% (33 of 113), and no staining in 45% (51 of 113). In the cases with both Nrp2 and CXCR4 positivity, co-localized expression of these two proteins was observed on sequential sections of the tumors (Figures 1C–D). As shown in Table 1, Nrp2 expression was correlated with lymph node metastasis (p = 0.0389), VEGF-C expression (p < 0.001), and cytoplasmic CXCR4 expression (p < 0.001).

### Nrp2 expression is correlated with patients' survival

**Figure 2:**
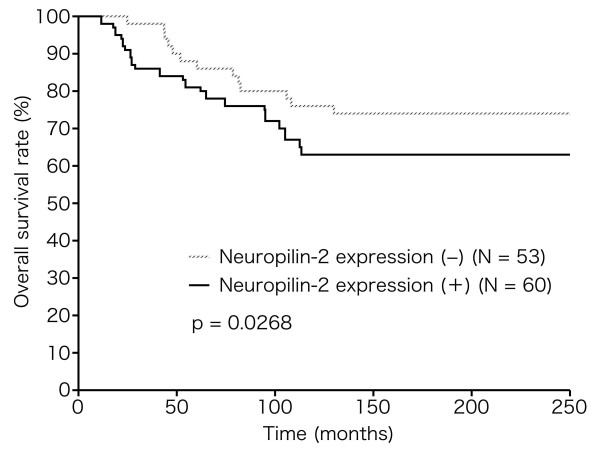
**Association of Nrp2 expression with prognosis of patients with breast cancer (Kaplan-Meier method and log-rank test)**. Nrp2 expression was significantly correlated to death (p = 0.0268).

Survival analysis was performed on 113 patients and the following variables were examined: Nrp2 expression, tumor size, lymph node metastasis, hormonal status, histological grade, VEGF-C expression, and cytoplasmic or nuclear CXCR4. As shown in previous reports [7,14], univariate survival analysis showed that tumor size, lymph node metastasis, ER status, VEGF-C expression, and cytoplasmic CXCR4 expression had significant prognostic value for DFS. Lymph node metastasis, VEGF-C expression, cytoplasmic CXCR4 expression, and also Nrp2 expression as shown in this study (p = 0.0268) had significant prognostic value for OS (Figure 2). Multivariate Cox regression analysis of all covariates focusing on DFS identified histological grade (p = 0.0099) as a significant independent prognostic factor, while both histological grade and Nrp2 expression were identified as independent prognostic factors for OS (histological grade, p = 0.0093; Nrp2 expression, p = 0.0453).

### Anti-Nrp2 blocks CXCR4 expression and CXCR4-induced migration

**Figure 3:**
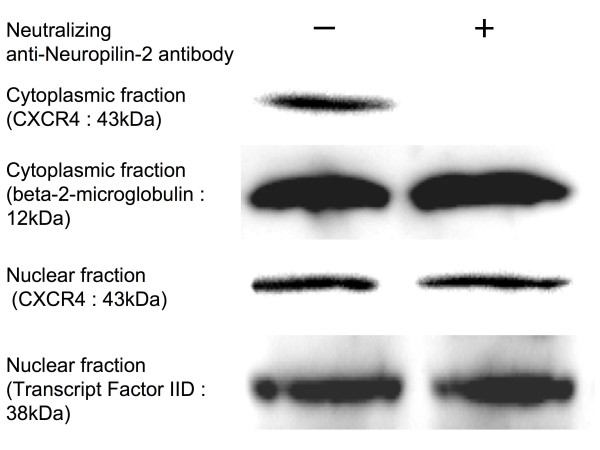
**Effect of neutralizing anti-Nrp2 antibody on CXCR4 expression**. MDA-MB-231 cells were treated with neutralizing anti-Nrp2 antibody and prepared for Western blot analysis. Treatment of the cells with anti-Nrp2 antibody significantly inhibited cytoplasmic CXCR4 protein expression. Nuclear CXCR4 protein expression was unchanged in this cell line.

**Figure 4:**
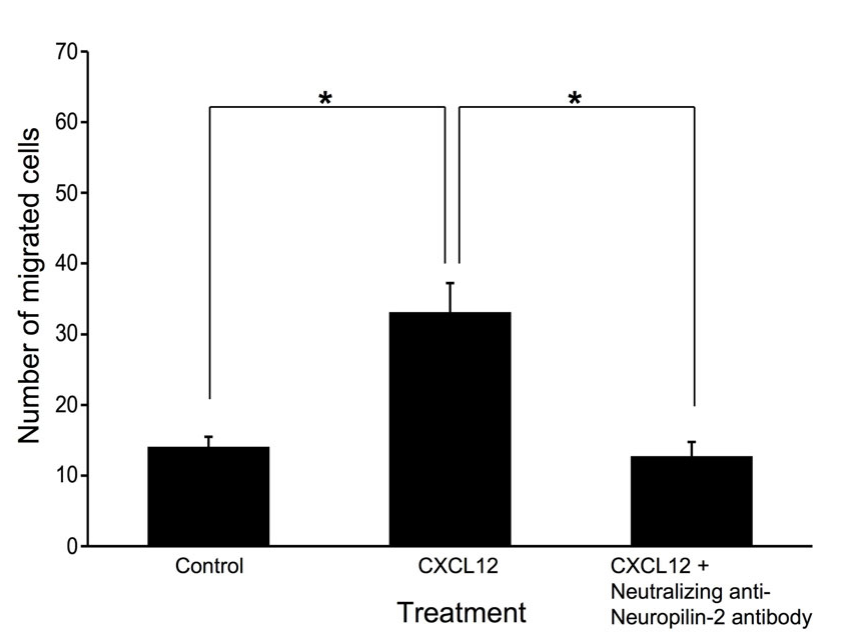
**Effect of neutralizing anti-Nrp2 antibody on CXCL12-induced chemotaxisis**. The chemotactic responses of MDA-MB-231 cells were significantly blocked by neutralizing anti-Nrp2 antibody. *Indicates significant difference (p < 0.05) from control and/or anti-Nrp2 antibody-treated cells.

As we had previously shown, endogenous cytoplasmic or nuclear CXCR4 expression was observed in the MDA-MB-231 cell line [14]. In this study, treatment of the cells with anti-Nrp2 antibody significantly inhibited cytoplasmic CXCR4 protein expression, although nuclear CXCR4 protein expression was unchanged in the treated cells (Figure 3). As shown in Figure 4, MDA-MB-231 cells showed significant chemotactic response to CXCL12. Furthermore, the chemotactic responses of MDA-MB-231 cells were significantly blocked by neutralizing anti-Nrp2 antibody.

## Discussion

The present study, to our knowledge, is the first one to demonstrate the clinicopathological significance of Nrp2 expression in human breast cancer. In normal breast epithelium, Nrp2 expression was not observed. In normal and cancer tissue, Nrp2 expression was identified in blood or lymph vessels. In addition to cytoplasmic VEGF-C and cytoplasmic CXCR4 expression, that had been already reported, Nrp2 expression was correlated with lymph node status and was positive in 53.1% of cases.

Previously, we reported that lymph node metastasis is correlated with cytoplasmic CXCR4 expression [14]. Given that Nrp2 expression was significantly correlated with cytoplasmic CXCR4 expression, we considered the possibility that Nrp2 expression with VEGF-C stimulation is involved in the expression of cytoplasmic CXCR4. In the present study, treatment of the MDA-MB-231 breast cancer cell line, which express Nrp2, endogenous VEGF-C, and cytoplasmic or nuclear CXCR4 [8,14,16], with anti-Nrp2 antibody significantly inhibited cytoplasmic CXCR4 protein expression. Furthermore, the chemotactic responses of MDA-MB-231 cells were significantly blocked by neutralizing anti-Nrp2 antibody. In these cells, cytoplasmic CXCR4 expression was unchanged after administration of recombinant human VEGF-C protein (wild type or Cys156Ser) (data not shown). Endogenous VEGF-C expression may be sufficient for endogenous CXCR4 expression, and Nrp2 with endogenous VEGF-C stimulation may regulate cytoplasmic CXCR4. Our data support the previous report that downregulation of Nrp2 in cancer cells can lead to fewer metastasis [20]. In addition, it is known that expression of CXCR4 in MDA-MB-231 cells is dependent on VEGF signaling [21]. Since the Nrp2 antibody blocked CXCR4 expression at 12 h after administration, the Nrp2 antibody may be affecting CXCR4 expression through the inhibition of VEGF signaling rather than inhibiting CXCR4 directly.

In this study, survival curves determined by the Kaplan-Meier method and univariate analysis demonstrated that Nrp2 expression was negatively associated with OS. Furthermore, multivariate analysis using the Cox stepwise regression analysis demonstrated that Nrp2 expression was still correlated with poor OS after consideration of other prognostic factors. Therefore, Nrp2 expression appears to be a reliable prognostic biomarker. Although we reported previously that cytoplasmic CXCR4 expression may become a useful prognostic indicator for OS, cytoplasmic CXCR4 expression was not identified as an independent prognostic factor for OS in this study (p = 0.0735). Considering that Nrp2 was correlated with poorer prognosis despite no correlation with distant metastasis, it may be necessary to increase the number of cases in the future study.

## Conclusion

Nrp2 expression is significantly correlated with VEGF-C expression, cytoplasmic CXCR4 expression, and lymph node metastasis in breast cancer. Nrp2 expression may serve as a significant prognostic factor for long-term survival in breast cancer. Nrp2 also regulates cytoplasmic expression of functional CXCR4 expression *in vitro*.

## Abbreviations

Nrp2: Neuropilin-2; VEGF: Vascular Endothelial Growth Factor; CXCR4: CXC chemokine receptor 4; ER: estrogen receptor; PgR: progesterone receptor; PBS: phosphate-buffered saline; DMEM: Dulbecco's Modified Eagle Medium; FCS: Fetal Calf Serum; DFS: disease-free survival; OS: overall survival.

## Competing interests

The authors declare that they have no competing interests.

## Authors' contributions

HY conceived the study, participated in the design of the study, conducted and evaluated both the immunostainings and the in vitro assay, performed the statistical analysis and drafted the manuscript. RK contributed to the design of the study, evaluated the immunostainings, and helped to draft the manuscript. MT, KY, HA, and MN participated in the design and coordination of the study. MI and TS contributed to the design of the study and interpretation of the results. YN participated in the design, evaluated both the immunostainings and the in vitro assay, and helped to draft the manuscript. All authors read and approved the final manuscript.

## Pre-publication history

The pre-publication history for this paper can be accessed here:

http://www.biomedcentral.com/1471-2407/9/220/prepub
